# Phylogenomic framework and virulence gene boundaries of emerging Shiga toxin-producing *Escherichia coli* O118 informed by the comprehensive profiling of 359 O118 genomes

**DOI:** 10.1080/21505594.2026.2672206

**Published:** 2026-05-15

**Authors:** Irvin Rivera, Sara S.K. Koenig, Armando L. Rodriguez, Joseph M. Bosilevac, Mark Eppinger

**Affiliations:** a Department of Molecular Microbiology & Immunology, University of Texas at San Antonio, San Antonio, TX, USA; b South Texas Center for Emerging Infectious Diseases (STCEID), San Antonio, TX, USA; c Research Computing Support Group, University of Texas at San Antonio, San Antonio, TX, USA; d U.S. Department of Agriculture (USDA), Agricultural Research Service (ARS), U.S. Meat Animal Research Center, Clay Center, NE, USA

**Keywords:** Shiga toxin-producing *Escherichia coli* (STEC), serogroups O118 and O151, whole genome sequencing, multi-locus sequence typing, comparative phylogenomics

## Abstract

Non-O157 Shiga toxin-producing *Escherichia coli* (STEC), particularly the O118 serogroup, are emerging pathogens linked to severe foodborne illnesses, which are characterized by the production of a potent phage-borne cytotoxin. This study explores the genomic landscape, virulence factors, and resistance traits of O118 STEC. We analyzed 357 publicly available O118 genomes, all but three available only in draft stage spanning ten different H-antigen types. To enhance the availability of high-quality reference genomes, we additionally included two O118:H16 STEC strains from our collection that were sequenced to closure. Multilocus sequence typing based on the 4,160 shared genes revealed phylogenetic clustering by H-type and delineated distinct STEC-phylogroups, alongside relationships to non-STEC pathovars such as uropathogenic *E. coli* (UPEC), enteropathogenic *E. coli* (EPEC), and enterotoxigenic *E. coli* (ETEC). Identified STEC-phylogroups encompass H6, H12, H16, and H2 strains with diverse Shiga toxin profiles (*stx*_*1a*_, *stx*_*2a*_, *stx*_*2b*_, *stx*_*2c*_, *stx*_*2f*_). A subset of H2-STEC lacked *stx*, suggesting potential secondary phage loss. Most STEC groups carried the locus of enterocyte effacement (LEE). Further, a strong correlation was observed between H-antigens and *eae* subtypes, with specific pairings such as H6/*eae-ι*, H16/*eae-β*, and H2/*eae-ε*. Horizontally acquired pathogenicity islands, including O-island 122 in H16 strains and a novel pathogenicity-associated island carrying antibiotic resistance, along with other loci related to colonization and interbacterial competition, further enhance their virulence potential. Our findings underscore the genetic diversity and virulence potential of O118 STEC. Understanding such phylogroup-linked virulence and resistance traits is crucial for effective surveillance and public health interventions.

## Introduction

*Escherichia coli* O118 is an emerging foodborne Shiga toxin-producing *E. coli* (STEC) serogroup with a bovine zoonotic reservoir [1]. Infections can cause severe human gastrointestinal disease and may progress to life-threatening complications, such as hemorrhagic colitis and hemolytic uremic syndrome (HUS) [2,3]. Vulnerable populations, such as children and the elderly, are particularly affected [4–6]. This underscores the importance of monitoring this emerging serogroup in both veterinary and public health contexts and assessing its virulence potential and phylogenetic boundaries [4,7]. *E. coli* are historically classified by their somatic O- and flagellar H-antigens [8–10]. Serotype O157:H7 is the dominant causative agent of STEC disease in the U.S [11,12]. However, the incidence of non-O157 infections has steadily increased in the U.S. and globally [13–16]. Emerging serogroups O26, O45, O103, O111, O121, and O145 account for most clinical non-O157 STEC infections in the US and are colloquially referred to as the “Big Six” [13,17,18]. These accounted for 83% of non-O157 STEC infections in the United States from 2000 to 2010 [19–22]. As diagnostic testing increased, non-O157 STEC, namely the “Big Six,” surpassed the previously dominant O157:H7 lineage in 2014 [15,18]. Between 2021 and 2023, non-O157 infections accounted for 168k annual cases, compared to 96k O157 cases [19,23]. The first reported human infection with *stx*_1_-positive O118:H2 STEC occurred in 1996 during a school outbreak in Japan [24] linked to salad [25]. Since then, strains of O118 (including its related O151 subgroup) [26,27] have been recognized as an emerging and human pathogenic STEC lineage, many of which possess Shiga toxins (*stx*), intimin (*eae*), and hemolysins [25,28]. The carried ФStx prophages feature different combinations of *stx*-suballeles [11,29] that can also form hybrid toxins [30]. Both *stx*_1_ and *stx*_2_ are potent translation inhibitors [25,29,31–33] and their alleles have been documented in O118 [6,28]. Stx_2_, particularly *stx*_*2a,*_ and *stx*_*2d,*_ have been associated with elevated cytotoxicity [34,35] compared to the morbidity caused by *stx*_*1a*+_ STEC [8,14,36]. However, Stx_1_ has been shown to exhibit increased cytotoxicity in Vero cells compared to Stx_2a_ [34,37,38]. Adding further complexity, Stx_1_ has been reported to reduce the overall toxicity of Stx_2a_, potentially through competitive binding or interference at the receptor level [34]. Besides the pathovar-defining Shiga toxin, another notable virulence factor includes the locus of enterocyte effacement (LEE). This pathogenicity island encodes a type III secretion system (T3SS) and effectors, the outer membrane adhesin intimin (*eae*), and its translocated receptor (*tir*) [9,25]. The latter is also carried by enteropathogenic *E. coli* (EPEC) [39]. Antimicrobial (AR) and multidrug-resistant (MDR) isolates are described for O118 STEC [25,28,40,41]. STEC also carry serogroup-specific virulence plasmids and an array of other plasmids with diverse functions [17,42,43]. Isolates belonging to serogroup O118 occur across multiple *E. coli* pathovars, including EPEC, which encode intimin and form characteristic attaching and effacing (A/E) lesions [4,24]; Enteroaggregative *E. coli* (EAEC) which form biofilm-like aggregates [4,13]; and extraintestinal pathogenic *E. coli* (ExPEC) that are capable of causing severe infections outside the gastrointestinal tract, such as urinary tract infections (UTIs) and bloodstream infections [24,44]. In this study, we provide a comprehensive framework for O118 *E. coli* with a focus on emerging STEC O118. The gathered information on virulence gene content and genome makeup provide a foundation to delineate evolutionary and pathovar boundaries of these emerging and human pathogenic non-O157 lineages.

## Materials and method

### Bacterial strains analyzed in this study

For this study, we analyzed a total of 359 *E. coli* O118 genomes: 357 publicly available genomes retrieved from the NCBI Pathogen Detection Isolate Browser (https://www.ncbi.nlm.nih.gov/pathogens/isolates/) as of July 2024, which included three closed genomes; and further two clinical O118 strains from the Nebraska Public Health Laboratory that we sequenced to closure to provide high-quality references. Strain-associated metadata and genome statistics are presented in Table S1.

### Genome sequencing, assembly, and annotation

Strains were cultured overnight at 37°C with shaking at 220 rpm in lysogeny broth (LB) (Thermo Fisher Scientific, Asheville, NC, USA). To maximize total genomic DNA (gDNA) yields, bacterial overnight cultures were diluted to OD_600_ of 0.03 in fresh LB medium and grown at 37°C with shaking at 220 rpm to mid-log phase (OD_600_ ~0.5). Total gDNA was extracted using the Monarch HMW DNA Extraction Kit (New England Biolabs, Ipswich, MA, USA). Genomes were sequenced to closure using Nanopore long-read technology (Oxford Nanopore, UK). Sequencing libraries were prepared with Rapid Barcoding Kit (RBK-114) according to the manufacturer’s instructions and sequenced on the PromethION platform on R10.4.1.Flow Cell (FLO-MIN114). Reads in the fastq format were imported into Galaxy v.22.05 [45]. Default parameters were used for all software unless specified otherwise. Fastq reads were QC-ed using FastQC (v.0.74+Galaxy0) (http://www.bioinformatics.babraham.ac.uk/projects/fastqc) and assembled with Flye (v.2.9.3) [46]. The resulting contigs were evaluated with QUAST (v.5.2.0 + Galaxy1). The chromosomal *dnaA* and plasmid *repA* genes were designated as the zero point of the closed molecules prior to annotation using the NCBI Prokaryotic Genome Annotation Pipeline (PGAP) [47]. Genome assemblies were profiled with SeqKit v2.8.2 to obtain genome statistics [48,49].

### Phylogenomic analyses and MLST-schemas

The O118 genomes, along with the sequence of K-12 substrain MG1655 [50,51], were imported into Ridom SeqSphere+ (v.8.3) (Ridom GmbH, Münster, Germany) for core genome (cg) and targeted Multilocus Sequence Typing (MLST) [52–54]. The Sequence Type (ST) was determined according to the EnteroBase schema [54]. Allele sequences for the Achtman scheme, targeting seven housekeeping genes (*adk*, *fumC*, *gyrB*, *icd*, *mdh*, *purA*, and *recA*), were accessed on the EnteroBase website (https://enterobase.warwick.ac.uk/species/ecoli/download_7_gene). A cgMLST schema was developed using the closed chromosome of K-12 substrain MG1655 [51] as seed as previously described [55]. Core and accessory MLST targets were identified according to the inclusion/exclusion criteria of the SeqSphere+ Target Definer. The allele information from the targeted seven-gene schema and the defined core genome gene of the panel strains were used to establish phylogenetic hypotheses using the minimum-spanning method with default settings [56,57].

### Pathogenome makeup and visualization

Chromosomes were compared with progressive Mauve [58] and visualized in pyGenomeViz-pgv-mauve [59] (https://github.com/moshi4/pyGenomeViz) using seaborn [60]. Chromosomes and carried plasmids were compared and visualized in Blast Ring Image Generator (BRIG) (v.0.95) [61]. Serotypes were determined *in silico* using *E. coli* Typer (ECTyper) (https://github.com/phac-nml/ecoli_serotyping) (Galaxy 1.0.0) in Galaxy v.22.05. If the *wzy* gene was reported absent by ECTyper, BLASTn was used to reevaluate its presence and fragmentation status. The ECTyper database was downloaded as a JSON file from GitHub (https://github.com/phac-nml/ecoli_serotyping) and converted into a blast database. The Average Nucleotide Identity (ANI) between all-vs-all 359 genomes was computed with skani [62], the distance matrix clustered by the UPGMA (Unweighted Pair Group Method with Arithmetic Mean) method and visualized using ANIclustermap (62) (https://github.com/moshi4/ANIclustermap). Virulence and antibiotic resistance genes (ARGs) were cataloged with Virulence Factor Database (VFDB) [63] and ResFinder (https://cge.cbs.dtu.dk/services/ResFinder/) [64], respectively. Pathovars were inferred *in silico* through BLASTp query [65] against a curated VFDB database, flagging *E. coli* pathovar-defining virulence genes as compiled from [63,66,67], e.g. EPEC (*eae*), STEC (*stx_1_* or *stx_2_*, ±*eae*), enterotoxigenic *E. coli* (ETEC) (*eltA*, *estA*, or *cfaB*), and uropathogenic *E. coli* (UPEC) (*fimH*). Analyzed O118 strains carry enterohemorrhagic *E. coli* (EHEC)-associated virulence factors (such as *stx*_2_ and *eae*). However, not all EHEC-genotype strains actually cause EHEC disease. We refer to them as STEC in this study to distinguish genetic potential from confirmed clinical disease manifestation, such as reported through patient data or outbreak reports. Ribosomal RNAs and CRISPR/Cas systems were detected with Basic Rapid Ribosomal RNA Predictor (Barrnap) [68] (v.0.7) and CRISPRCasFinder [69] (v4.2.20) in Proksee [70] (v.1.1.0). Anti-phage systems were detected using DefenseFinder [71] (Galaxy v.1.3.0+galaxy0). Prophages were compared and visualized in Easyfig (v.2.2.2) [72]. Boundaries and locations of intact, partial, or remnant prophages were identified using PHASTEST [73], followed by manual curation of the ФStx-prophage and core genome borders by Mauve alignment of the genome to *E. coli* K-12 substrain MG1655 (GenBank accession U00096) [50] in Geneious Prime (v.2024.0.5). Direct repeats caused by phage integration were identified by BLASTn self-alignment and the Repeat Finder plugin (v1.0.1) in Geneious Prime. Replicase subtyping was performed to characterize the ФStx_1a_-encoding prophages, following the typing scheme introduced by [74]. Replicases were typed with EHEC phage replication unit (*eru*) schema [75,76]. The subtypes of phage-borne *stx* were determined by BLASTn against a curated *stx*-suballele database, as previously described [17,77,78]. A hierarchical classification approach was implemented to categorize phage-associated genes from the PHASTEST JSON output. All present phage-associated genes were further curated processing type and name descriptors, along with reported Gene Ontology (GO), for more in-depth functional classification. Genomic islands (GI) were detected with IslandViewer4 [79–81]. LEE islands and boundaries were curated, guided by LEE1-LEE4 operon genes *espG and espF*, respectively, and visualized in EasyFig (v.2.2.2) [72]. Intimin subtypes were determined by BLASTn against a curated database of published *eae*-subtype sequences, as reported previously [17]. Transposable elements and Insertion Sequence (IS) elements, along with inverted repeats, were cataloged through BLASTn [82] against the Transposon Central (TnCentral) database [83], comprised of TnCentral+Integrall+ISFinder entries [84,85] and ISEScan (v.1.7.2.3+Galaxy0) [86].

### Plasmid makeup and visualization

Plasmids were visualized and decorated in BRIG (v.0.95) [61]. Plasmid mobility and incompatibility groups were recorded with Mobilome Typer (MOB-Typer) (v.3.0.3+Galaxy0) [87] and ABRicate (Galaxy v.1.0.1; https://github.com/tseemann/ABRicate) with options --minid 95 --mincov 90 using the PlasmidFinder database [82,88] (Carattoli A, Zankari E, Garcia-Fernandez A, Volby Larsen M, Lund O, Villa L, Aarestrup FM, Hasman H. Antimicrob. Agents Chemother. 2014. April 28th.). Using a MASH-based search strategy, phylogenetically related plasmids were identified by comparison against the PLSDB database (v.2024.05.31.v2) [89,90]. Serotypes of the plasmid-associated chromosomes molecule were determined *in silico* using ECTyper (https://github.com/phac-nml/ecoli_serotyping) (Galaxy v1.0.0) in Galaxy v.22.05. Virulence genes were cataloged with VFDB [63]. Bacteriocins, ribosomally synthesized and post-translationally modified peptides (RiPPs), were recorded with BAGEL4 (http://bagel4.molgenrug.nl/) [91]. Genomic islands were detected with IslandViewer4 [79–81]. Mobile Genetic Elements (MGEs) were identified in Proksee [70] based on homology to entries in the mobile orthologous groups database (mobileOG-db) [92]. Transposable elements and IS elements were identified with ISEScan (v.1.7.2.3+Galaxy0) [86] and by BLASTn queries [82] against the TnCentral database, comprised of TnCentral+Integrall+ISFinder entries [83–85].

### Antimicrobial susceptibility testing

Susceptibilities were determined as described previously [93]. Broth microdilution was performed using the SENSITITRE™ NARMS Gram Negative panel (Fisher Scientific, Cleveland, OH, USA) then output data were interpreted using CLSI M100 (CLSI, 2022) guidelines fitting the National Antimicrobial Resistance Monitoring System (NARMS) standards [94]. The antimicrobial agents included in the panel, along with their abbreviations and resistance breakpoints, were as follows: amikacin (AMI), ≥64 μg ml^− 1^; amoxicillin – clavulanic acid (AMC), ≥32/16 μg ml^− 1^; ampicillin (AMP), ≥32 μg ml^− 1^; cefoxitin (FOX), ≥32 μg ml^− 1^; ceftiofur (TIO), ≥8 μg ml^− 1^; ceftriaxone (AXO), ≥16 μg ml^− 1^; chloramphenicol (CHL), ≥32 μg ml^− 1^; ciprofloxacin (CIP), ≥4 μg ml^− 1^; gentamicin (GEN), ≥16 μg ml^− 1^; kanamycin (KAN), ≥64 μg ml^− 1^; nalidixic acid (NAL), ≥32 μg ml^− 1^; streptomycin (STR), ≥64 μg ml^− 1^; sulfisoxazole (FIS), ≥512 μg ml^− 1^; tetracycline (TET), ≥16 μg ml^− 1^; and trimethoprim – sulfamethoxazole (COT), ≥4/76 μg ml^− 1^. Resistance to three or more antimicrobial classes was classified as MDR. The antimicrobial classes represented in this panel were: aminoglycosides (AMI, GEN, KAN, STR), β-lactam/β-lactamase inhibitor combination (AMC), cephems (FOX, TIO, AXO), folate pathway inhibitors (FIS, COT), penicillin (AMP), phenicol (CHL), quinolones (CIP, NAL), and tetracycline (TET).

### Supplemental text

#### Plasmid profiles of serogroup O118 *E. coli*

The 359 analyzed closed and draft genomes identified 36 replicons, which provided a testament to the plasticity of plasmids in this group. Among these were incompatibility group families associated with virulence plasmids and colicins (Table S2). The most prevalent replicon in STEC O118 was IncFIB (AP001918) of H2 strains (97.1%, 235/242) and of H16 strains (94.6%, 88/93). These usually 100 kb or larger low-copy plasmids have been associated with disseminating virulence genes in *Enterobacteriaceae* [95]. Other Inc-types with shared distribution were colicin-type Col156, present in H2 (42.6%, 103/242) and H16 (7.5%, 7/93) strains, as well as present in the pO111 plasmid in H16 (29.0%, 27/93) and H2 (14.5%, 35/242) strains. The presence of Col156 is often associated with plasmids that also carry antibiotic resistance genes [96]. Carriage of virulence plasmid pO111 has been linked to severe gastrointestinal disease [97]. Another noteworthy observation was that most STEC H16- and H2-phylogroups feature IncB/O/K/Z_3 groups (95.7% and 90.7%), and IncFII(pCoo) that is present in all H11-strains. It would be valuable to correlate plasmid replicons with AMR and virulence genes. Unfortunately, the majority of O118 genomes are in draft stage, which limits the ability to unambiguously associate gene inventories with the multiple replicons identified in a given strain. The inclusion of closed genomes in future studies will be essential to resolve plasmid structures and enable the accurate mapping and correlation of AMR and virulence determinants.

## Results and discussion

### Genomes of closed STEC O118

**Figure 1.**
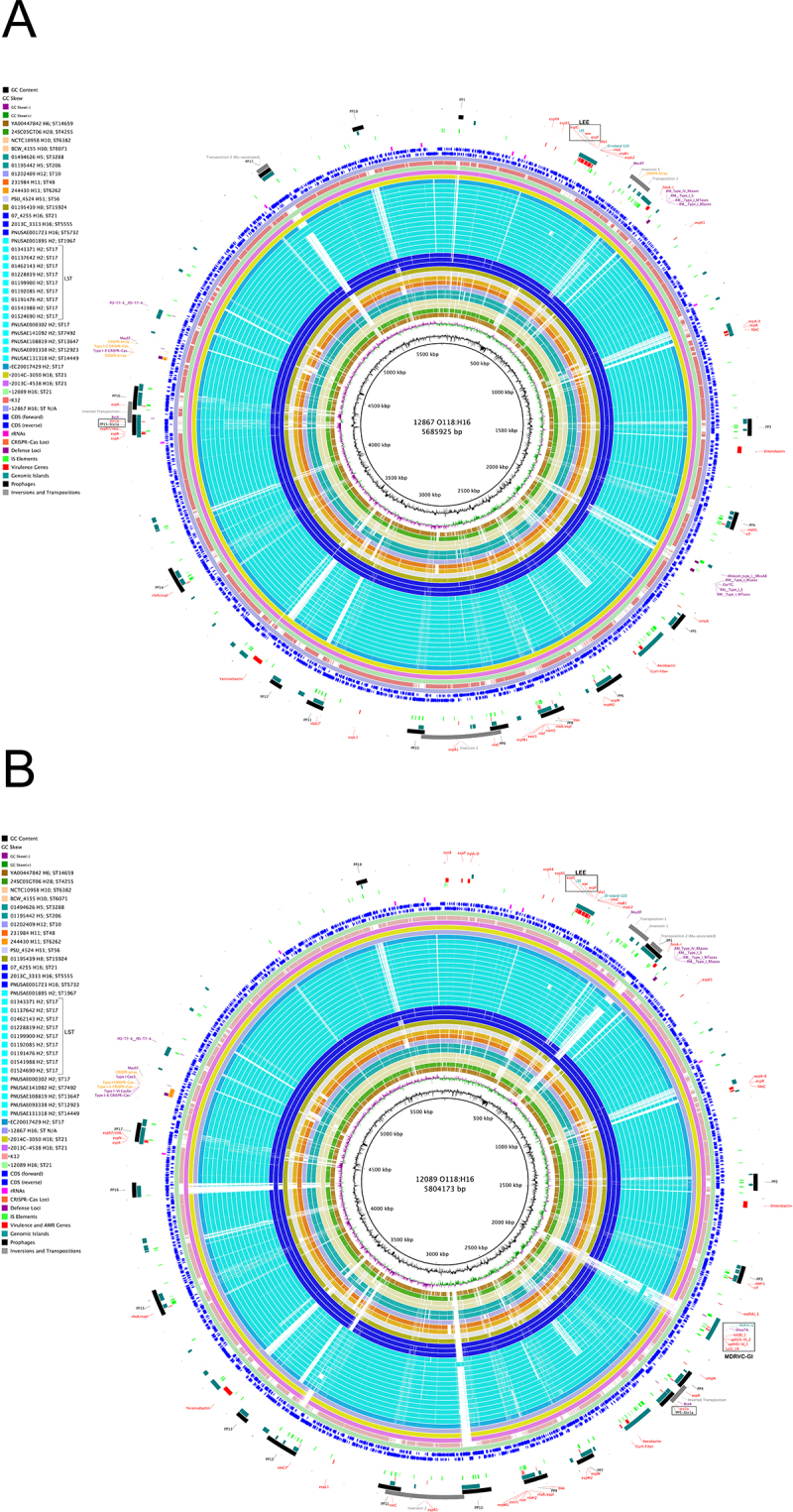
Comparison of O118 STEC chromosomes. BRIG comparison of representative genomes for all identified STEC-phylogroup, considering H-antigen and sequence type, referenced to O118:H16 strains A) 12867 and B) 12089. *E. coli* K-12 is also included to highlight STEC-specific genome content. Query genomes are color-coded, and their order reflects the inferred phylogenomic relationships. Coding sequences are shown as arrows on the + and – strands, and functional annotations for virulence genes and other loci of interest are highlighted.

**Figure 2.**
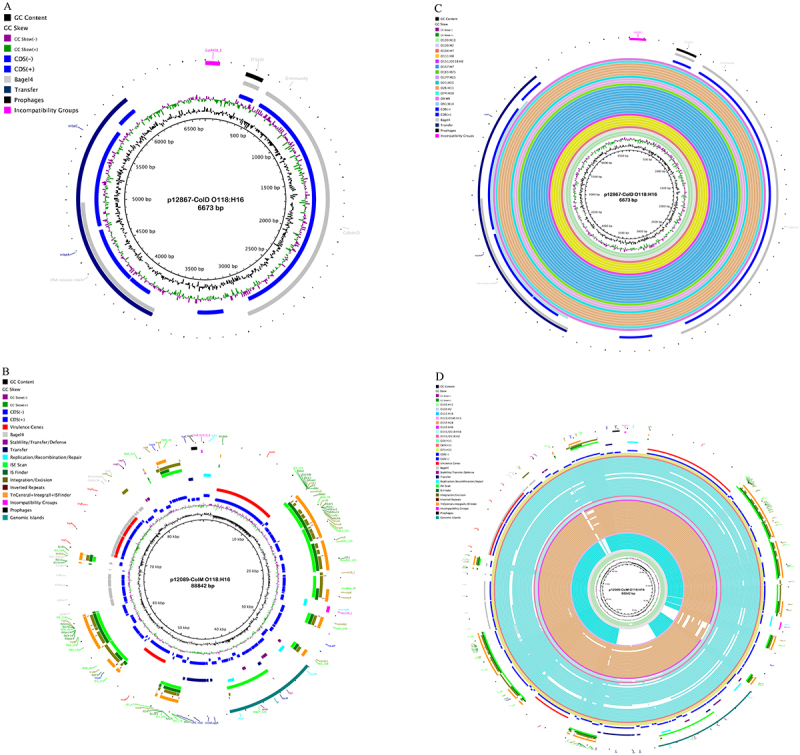
Plasmid inventory of the two sequenced O118 STEC strains. (A) The small 6,673 bp ColD-colicin plasmid in H2-strain 12867, (B) the 88,842 bp multifunctional ColM-colicin and virulence plasmid of H16-strain 12809 features an array of virulence and conjugation loci, (C), and (D) phylogenetically related plasmids were detected in diverse STEC serogroups.

At the time of data collection, three closed serogroup O118 genomes were publicly available, all of which are STEC: H6-strains 2013C-4538 and 2014C-3050 from human stool [98] and H2-strain EC20017429 isolated in 2017 from a gastroenteritis patient [99]. To support comprehensive analyses of O118 STEC, we sequenced two clinical H16-isolates, strains 12089 and 12867, to closure using long-read sequencing [17,100,101]. This approach yielded high-quality closed chromosomes of 5,804,173 and 5,685,925 bp and recovered carried plasmids (Figures 1, 2). Genome statistics and strain-associated metadata are provided in Table S1. The average nucleotide identity processing closed and draft O118 genomes was 99.34%, indicative of the highly conserved chromosomal *E. coli* backbone [102,103] (Figure S1).

### Mobilome of closed STEC O118 genomes

Mobile genome elements are major drivers of pathogenome diversification [32,104,105]. The identified prophages, GIs, and IS elements contributed significantly (24.11 to 28.51%) to the genome content of the closed O118 STEC, comparable to observations in other STEC-lineages [105,106] (Table S2). These STEC strains contained about 1 Mb of additional sequence information compared to nonpathogenic *E. coli* strain K-12 substrain MG1655 [50], included in this comparison. These included pathovar-defining ФStx prophages [25,29,31–33,107–109] and other virulence hallmarks, such as the LEE Pathogenicity Island [25,110,111]. IS elements have been utilized to delineate distinct phylogenetic lineages in STEC [17,112–114]. We recorded IS element prevalence and types in the five closed genomes (Figure S2), showing both shared (IS3_168) but also H-antigen-specific IS elements (e.g. IS3_255, IS4_463).

### Large chromosomal rearrangements in H16-strains 12867 and 12089

Mauve comparison of closed O118 chromosomes revealed largely genome-wide synteny, though we observed large chromosomal rearrangements (LCRs). LCRs can generate rapid phenotypic changes despite identical gene content by modifying chromosome organization [115] and have been associated with environmental fitness, antimicrobial susceptibility, type III secretion, and Stx production [116–118]. As evident in Figure 1 and S3, the inversion and transposition events that differentiate the genomes of the sequenced H16-strains 12867 and 12089 were associated with adjacent or flanking mobility or ribosomal RNA loci [119–122]. Two rearrangements are linked to prophage regions, in similarity to LCRs reported in STEC O157 [118,123], and both inversion 1 and transposition 1 are associated with IS-elements (Figure 1).

### Colicinogenic and virulence-associated plasmids in H16-strains 12867 and 12089

The sequenced H16-strain 12867 and H16-strain 12089 are both colicinogenic (Figure 2). Colicins are produced and toxic to *E. coli*, and support the ability of *E. coli* to compete for a shared niche [124]. Plasmid p12867-ColD [124], showed the typical organization into D-lysis, D-immunity, and Colicin D [125], along with a mobilizable DNA-relaxase *mbeA* [126]. Additionally, the plasmid-borne SOS inhibition gene *psiB* was detected [127]. A MASH-based survey identified related plasmids in STEC O157:H7, the “Big Six” serogroups, and other STEC-associated serogroups (Table S2). The larger 88 kb plasmid p12089-ColM encodes Colicin M and its associated M-immunity protein, its other gene content identified it as a virulence plasmid. Notable were hemolysin (*hlyCABD)* [128], serine protease (*espP)* [129], fimbrial regulator *fimB*, and the STEC adherence factor *toxB* [130]. Additionally, modulators of lipopolysaccharide and exopolysaccharide biosynthesis (*lpxM*, *icaB*) [131,132] along with SOS-inhibition gene *psiB* [127], were also present. Approximately 43% of the plasmid sequence are mobility and conjugation loci [119,133]. A MASH-based plasmid survey identified related plasmids among H16 and H2, Big Six and other STEC serogroups [134–136] (Table S2).

### Comparison of ФStx prophages in closed O118 STEC

**Figure 3.**
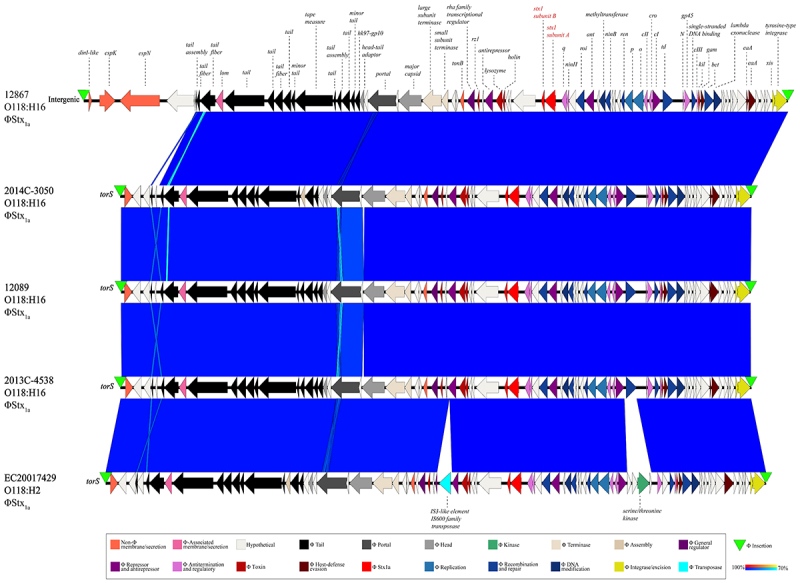
Comparison of ФStx-prophages in closed O118 STEC. BLASTn-based comparison of the ΦStx_1a_ prophage architectures and gene content. The comparison shows that ΦStx1a phages show considerable genomic plasticity and are inserted at *torS* or intergenically close to the *dinI* locus.

Comparative genomics provided insights into the shared and specific virulence traits in the identified STEC-phylogroups. Bacteriophages target conserved chromosomal loci and undergo repeated acquisition, loss, and microevolution, thereby collectively shaping bacterial gene content and pathogenicity traits [104,137,138]. A comparison of the complete ФStx_1_ prophages extracted from the closed genomes identified two insertion sites: *torS* and an intergenic insertion upstream of *dinI* with the phage boundaries marked by flanking direct repeats (Figure 3, Table S2). The *torSTR* locus was one of four identified ФStx_1a_ integration sites in O26:H11 ST21 [139], an intergenic attachment site common in *E. coli* [140]. The ФStx_1a_ prophages show an overall high-degree of sequence identity and synthetic organization. The *torS* ΦStx_1a_ phage of the H2-strain EC20017429 was distinguished by the presence of an IS3-like element and cluster of three hypothetical genes and a kinase, absent in the other H16 strains. The larger ΦStx_1a_ of H16-strain 12867 carried a DUF550 protein, type III effectors EspK and EspN, and a hypothetical protein.

### Locus of enterocyte effacement pathogenicity island in closed O118 STEC

**Figure 4.**
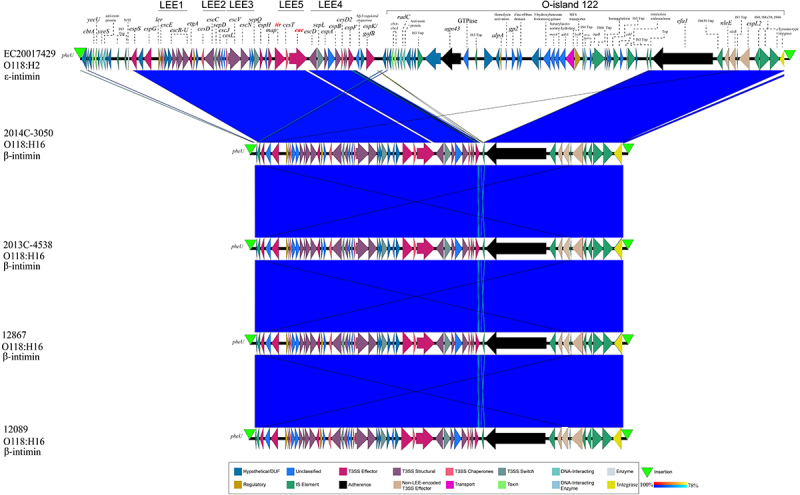
LEE islands of closed O118 STEC. BLASTn-based comparison of LEE islands inserted into the *pheU*. The organization of the LEE1 to LEE5 operons and O-island 122, disrupting the LEE island in H2-strain EC20017429, is indicated. The order of strains reflects their inferred phylogenomic position.

The LEE pathogenicity island is carried by diverse STEC serogroups, forming characteristic attaching and effacing A/E lesions [141–143]. The comparison of LEE islands extracted from the closed O118 STEC genomes (Figure 4) showed they were highly conserved and organized in five polycistronic operons (LEE1 to 5). These encoded T3SS regulators and effectors [144]. All were integrated at *pheU* tRNA, a known LEE integration site in *E. coli* [145–147]. The H2 strain was distinguished by an adjacent O-island 122 within the LEE island boundaries marked by 17 bp direct repeats (Table S2). This island encodes non-LEE-encoded (Nle) effectors and transport and regulatory elements that enhance *E. coli* pathogenicity by promoting intracellular survival, adhesion, immune modulation, and enterotoxin production [148,149]. Its carriage has been associated with highly virulent STEC, causing HUS [149,150]. The H16 phylogroup, represented by strains 2014C-3050, 2013C-4538, 12867, and 12089 features the β-intimin subtype, whereas the ε-intimin subtype is present in the H2 strain EC20017429 (Figure 4).

### Pathogenicity associated multi-drug resistance, virulence, and competition island

**Figure 5.**
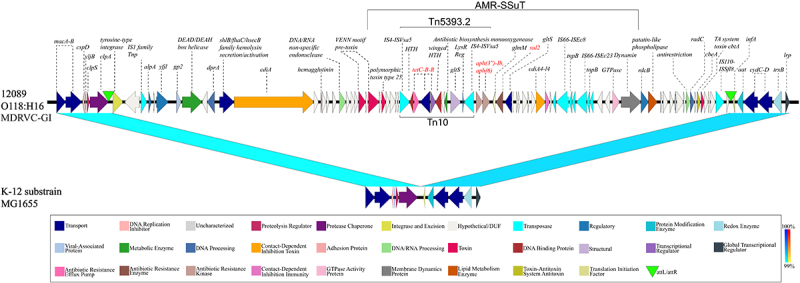
Modular architecture of the MDRVC island. This newly discovered 74kb multifunctional island, inserted into the *tRNA-ser* of H16-strain 12089, features a complex modular architecture. It integrates an antimicrobial multi-drug resistance cassette with gene loci, promoting virulence, interbacterial competition, stress resilience, and overall fitness.

In strain H16- 12089, a novel 74 kb Resistance, Virulence, and Competition (MDRVC) Island with a complex modular architecture was discovered inserted into tRNA-Ser (Figure 5). This island featured a tyrosine-type integrase [151], with borders marked by direct repeats [152] (Table S2). A core component of this island was the MDR-element AMR-SSuT, which has been previously described in STEC O157, though the genomic context was unclear [153]. This island carries tetracycline, streptomycin, and sulfonamide loci. Embedded within this element was streptomycin resistance transposon Tn5393.2 [154], itself disrupted by an ISVsa5-flanked Tn10 [155], introducing tetracycline [156,157]. The *sul2* locus, however, is located outside this Tn5393.2-Tn10 composite, proximate to IS66-like elements, previously linked to antimicrobial resistance in *E. coli* [158]. Prominently featured were genes that aid interbacterial competition, such as a *cdiA*-mediated contact-dependent inhibition [159] associated with VENN motif pre-toxin [160] and the *cbtA-cbeA* toxin-antitoxin system [161]. A broad array of virulence loci was cataloged; among these was the hemagglutinin adhesin [159] and the hemolysins secretion gene (*fhaC*), which likely interacts with the hemolysin on p12089-ColM (Figure 2). Further diverse regulator genes were associated with stress-response. This included *lysR* [162], a DEAD/DEAH-box helicase [163], *alpA* [164], and a bacteriophage defense gene *gp2* [165], along with membrane modeling genes, patatin-like phospholipases and dynamin-like protein *rdcB* [166,167]. DNA-modification enzymes support genomic integrity through repair (*radC*) or endonuclease-mediated defense [168,169]. Altogether, the carriage of this multifunctional GI by strain 12089 suggests increased pathogenic potential and fitness, promoting virulence, antimicrobial resistance, stress adaptation, and also genomic stability.

### Phylogenomic framework and pathovar boundaries of serogroup O118/O151 STEC

**Figure 6.**
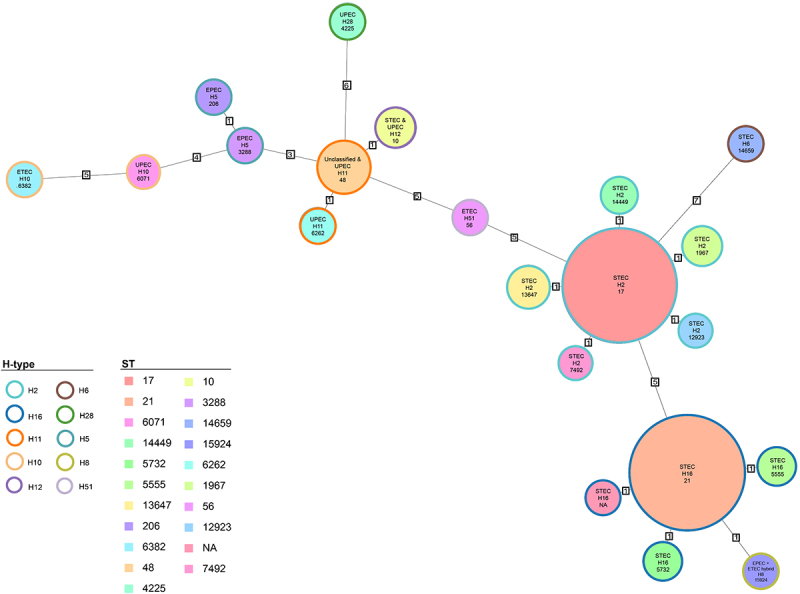
MLST-inferred phylogeny of serogroup O118. The relatedness of O118 strains was determined using MLST in ridom SeqSphere+ through both targeted seven-gene MLST accessed in the EnteroBase website. Numbers on connecting branches indicate the number of genes with differing allele status. The shared gene inventory of 4,160 genes comprises 2,437 core and 1,723 accessory loci according to the inclusion/exclusion criteria of the SeqSphere+ target Definer. Circle size corresponds to the number of isolates with identical st.

**Figure 7.**
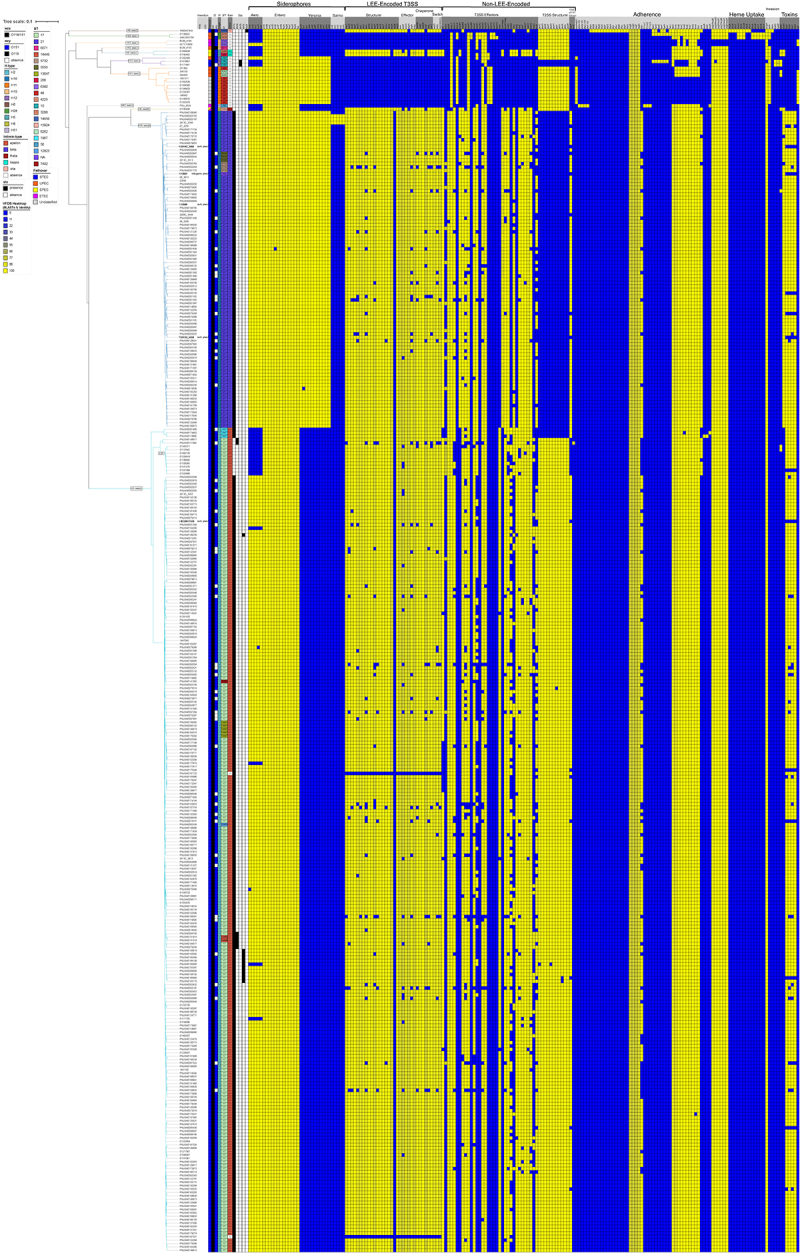
Phylogeny and distribution of virulence genes. The relatedness of analyzed O118/O151 strains was determined using cgMLST in ridom SeqSphere+ using the closed chromosome of *E. coli* strain K12 substrain MG1655 as seed, complemented by the 357 publicly available strains and two closed genomes strains 12089 and 12867. The shared gene inventory was determined at 4,160 genes. These comprise 2,437 core and 1,723 accessory loci according to the inclusion/exclusion criteria of the SeqSphere+ target Definer. The topology and clusters indicate a strong association between H-antigen, *eae*-subtype, and st in LEE+ isolates. A total of 199 distinct virulence genes were cataloged, of which 16 are shared. The STEC-pathogroups are comprised of H-antigens 6, 12, 16, and 2 and carry the *stx*_1a,2a,2b,2c,2f_ suballeles.

To support broad-scale analysis, we included all publicly available serogroup O118, O151, or O118/O151 genomes, as identified in the NCBI Pathogen Detection Isolate Browser, for which a common ancestry has been proposed [170]. O118 and O151 strains are differentiated by subtle antigenic changes in their O-antigen gene clusters (OGCs). Their OGCs share > 99% nucleotide sequence identity and differ by only two nonsynonymous substitutions (O118 → O151): a V292A substitution in *wzy* and a P88S substitution in *fnlB*. To comprehensively capture this diversity, we included all genomes annotated as O118/O151, O118, or O151 in our analyses. Notably, 358 of 359 analyzed genomes possessed the O151-*wzy* variant, while a single strain carried O118-*wzy* allele (Table S1) [170–172]. Strain-associated metadata and genome statistics of this expanded strain panel can be found in Table S1. This panel of 359 O118 genomes features ten H-antigens, as determined by *in silico* flagellin (*fliC*) subtyping [173,174]. The shared gene inventory was determined at 4,160 genes, comprised of 2,437 core and 1,723 accessory loci, indicative of the genome plasticity found in heterogenous O118 serogroup *E. coli* (Figure 7, Table S4). The ANI of 96.32% was computed through an all-vs-all comparison of the 359 O118 genomes. The UPGMA-clustering of ANI values recovered the different H-phylogroups (Figure S1). The majority belonged to H2 (*n* = 242), followed by H16 (*n* = 93) and H11 (*n* = 11), with relatively few available representative genomes of H10 and H12 (*n* = 3), H5 and H28 (*n* = 2), and a single strain for H6, H8, and H51 (Table S1). To investigate the phylogenomic and virulence boundaries of emerging O118 STEC among the heterogenous serogroup O118/O151 strains, we established a phylogenomic framework informed by targeted and core genome MLST (Figure 6, 7). The tree topology partitions the isolates by H-antigen and ST. Pathovars were inferred *in silico* from their VFDB virulence gene profiles. The STEC phylogroups among serogroup O118 were comprised of H2, H6, H12, and H16 strains. Non-STEC pathovars belonged to EPEC (H5), ETEC (H10, H51), UPEC (H28, H10, H12, H11), unclassifiable (H11), and a single strain containing virulence genes associated with both ETEC and EPEC (Figure 7, Table S1, Table S3). Notably, H10 antigen belonged to EPEC and UPEC, which formed distinct clusters, also evident in the ANI heatmap (Fig. S1).

### Correlation between stx status, H-antigens, and intimin subtypes

For STEC, the following *stx/eae*-genotype profiles were recorded: H6 (*stx*_*2f*_, *eae-ι*), H12 (*stx*_*2b*_, *eae*(-)), H16 (*stx*_*1a*_, *eae-β*) and H2 (*stx*_*1a*_, *eae-ε*; *stx*_*2a*_, *eae-ε*; *stx*_*1a*_, *stx*_*2c*_, *eae-ε*; and *stx*_*1a*_, *stx*_*2a*_, *eae-ε*) [77]. The best-represented STEC groups in the analyzed strain panel were H16 (*n* = 93) and H2 (*n* = 242). H16 STEC was genetically uniform (*stx*_*1a*_, *eae-β*) compared to H2 STECs with significant toxicity profile heterogeneity. Among H2 STEC, the predominant *stx* genotype was *stx*_*1a*_ only (*n* = 215), followed by *stx*_*1a*_ + *stx*_*2c*_ (*n* = 11), *stx*_*1a*_ + *stx*_*2a*_ (*n* = 5), and *stx*_*2a*_ only (*n* = 2). Within the dominant H2 STEC, we further identified a distinct *stx*(-) sublineage. Further analyses of the H2 ΦStx_1a_ phage insertion site at *torS* (Figure 3) detected phage remnants (Figure S4). We thus speculate that this lineage evolved from ST-17, *stx*_*1a*_*+* ancestors and represents Lost Shiga Toxin (LST) isolates [100]. Their closest relatives were *stx*_*2a*_*+* strains that similarly lack *stx*_*1a*_*+*. However, the draft status of these genomes hindered further investigation into the ФStx insertion locus at *torS* to evaluate the ancestral or evolved ФStx-status (Figure S4). We thus speculate that this STEC lineage may have secondarily acquired *stx*_*2a*_, essentially transitioning between *stx*(+/−) states [100,175,176]. Similarly, the LEE-H12-group contained both *stx*_*2b*_*+* and *stx-* isolates. However, the draft status of the genomes hindered further investigation of the ФStx status.

Except for the H12 STEC, the LEE island is present in all other identified STEC phylogroups (H2, H6, H12, and H16) (Figure 7). The LEE island is also present in EPEC (H5) and a strain containing genes associated with both ETEC and EPEC (H8) (Figure 7). Over 20 intimins mediating the attachment to intestinal cells are currently described [17]. Previous studies indicated a correlation between the H-antigen and the intimin subtype in LEE-positive *E. coli* [177]. Indeed, we found a correlation between H-antigens and the five *eae-*suballeles present in serogroup O118. The intimin *ε*-subtype is associated with H2 STEC, exemplified by strain EC20017429, and LST strains of this group (Figure 7). This group also contains *eae*-negative strain PNUSAE161722, missing integral LEE components and effectors (Figure 7). Its position in the tree and intimate relationship to ST-17 LEE+ isolates may suggest secondary loss rather than an ancestral LEE-status. The H16-phylogroup features the *β*-intimin, represented by strains 2014C-3050, 2013C-4538, 12867, and 12089 (Figure 4). We further observed the following correlations H6-*eae-ι*, H5-*eae-κ*, and H8-*eae-θ*, in the other LEE+ phylogroups, though these were represented by only a few strains.

### Antimicrobial- and multidrug-resistance loci in serogroup O118

**Figure 8.**
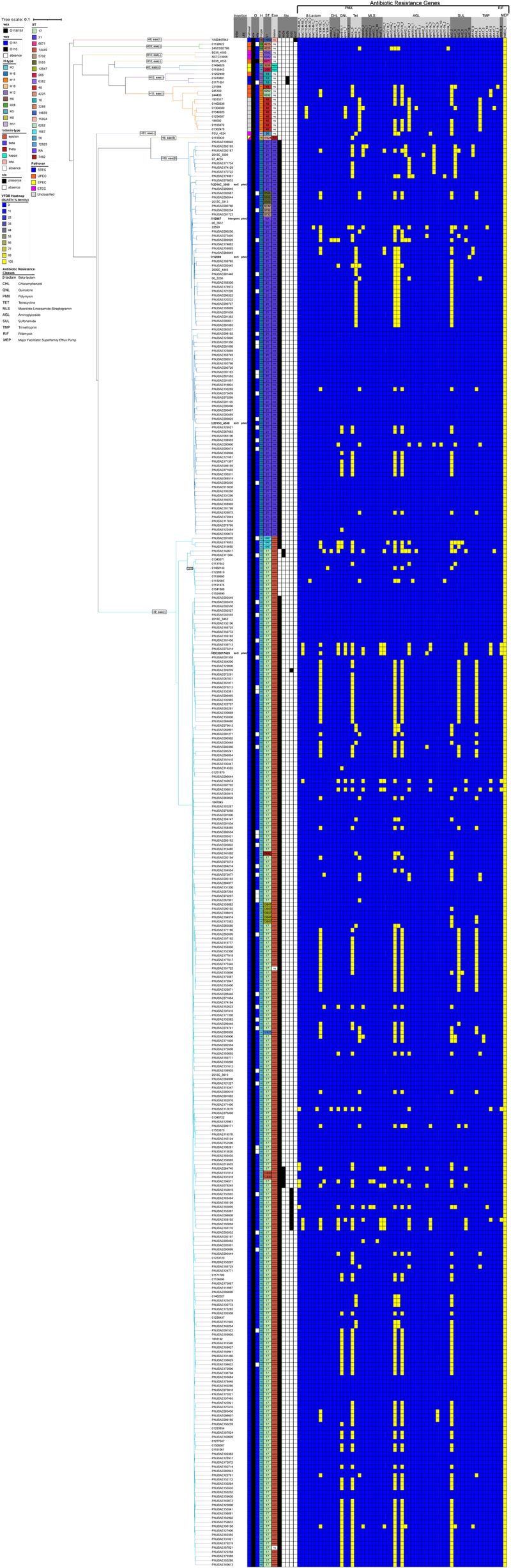
Phylogeny and distribution of antibiotic-resistance genes. Percentage identities for each antibiotic-resistance gene identified in ResFinder are visualized in a heatmap. The strains feature a total of 58 antibiotic-resistance genes that were classified into 10 antibiotic classes.

AR and MDR *E. coli* O118 STEC strains have been previously reported [41,99]. Consistent with these observations, AR/MDR strains were detected within STEC phylogroups H16 and H2 (Figure 8), and not in other STEC lineages. Among the resistant O118 STEC, we identified genes associated with resistance to ten antibiotic classes: β-Lactam, Chloramphenicol (CHL), Quinolone (QNL), Tetracycline (Tet), Polymyxin (PMX), Macrolide – Lincosamide – Streptogramin (MLS), Aminoglycoside, Sulfonamide, Trimethoprim, and Rifampin (RIF). Numerous MDR isolates, as defined by resistance to three or more antibiotic classes, were detected. A prominent three-class profile – tetracyclines, aminoglycosides, and sulfonamides – was observed frequently in H16 and H2 STEC, similar to the one observed in the MDRVC-Island of strain 12089 (H16, ST21) (Figure 5). Antimicrobial susceptibility testing showed that the 12089 isolate was resistant to streptomycin (≥32 µg/mL), sulfisoxazole (≥512 µg/mL), and tetracycline (≥16 µg/mL). We also noted that resistance gene presence and combinations followed phylogroup-specific patterns, as evident from the cgMLST-based phylogeny (Figure 8). However, due to the draft status of most genomes, we were limited in our ability to determine the genomic context, whether these AR loci are chromosomal (Figure 5), plasmid-borne [99], or phage-associated [12] (Figure 8).

## Conclusion

Whole-genome sequencing (WGS) typing strategies have significantly advanced our understanding of the genomic makeup and evolution of *E. coli* pathovars [11]. The high-resolution framework established for the *E. coli* serogroup O118/O151 complex allowed us to define the phylogenomic boundaries and virulence traits of emerging O118 STEC. For partitioning O118 serogroup isolates, their H-antigen was a robust phylogenetic marker [178], although genetically unrelated *E. coli* strains may share H-types due to lateral acquisition [179]. Among the delineated STEC O118 phylogroups, we observed substantial plasticity in virulence and resistance inventories, characterized by diverse *stx* and *eae* suballeles. The presence or absence of *stx* was shaped by dynamic ΦStx prophage acquisitions but did not necessarily reflect evolutionary relationships [176]. Notably, we identified a secondary Stx prophage loss event in an *stx*-negative H2 subgroup branching from ST-17 STEC, suggesting that such losses, followed by potential reacquisition, may lead to transitional shifts in pathogenic potential [100]. Our findings showed a strict correlation between the H-antigen and LEE-borne intimin subtype in STEC (H6/*eae-ι*, H16/*eae-β*, H2/*eae-ε*) and non-STEC (H5/*eae-κ*, H8/*eae-θ*) isolates, reinforcing the hypothesis of evolutionary lineage sorting [177,180]. We identified horizontally acquired pathogenicity-associated islands (PAI) that enhance virulence potential. Notable was the O-island 122 inserted into the LEE-island of clinical H2-strain EC20017429, a locus previously associated with severe disease outcomes [148–150]. The discovered multifunctional island in clinical H16-strain 12089 enhances interbacterial competitiveness, virulence, stress adaptation, and may increase the strain’s fitness and pathogenic potential. However, disease outcome is multifactorial, resulting from the complex interplay between the infective agent, the host microbiota [181,182], and the infected individual [183,184], and thus cannot be reliably predicted *in silico*. The emergence of clinically relevant O118 STEC in both human and animal reservoirs, with the potential to cause severe foodborne disease, highlights the need for ongoing genomic surveillance. Insights into pathogenome plasticity and evolutionary trajectories, gained through comparative genomic approaches, are critical for informing public health strategies, risk assessment, and infection control efforts.

### Implications for public health and risk assessment

By linking phylogroup structure to virulence and AMR inventories, this work aids in the phylogenetically informed surveillance, risk assessment, and outbreak investigations of emerging O118 lineages. The established phylogroup-linked and gene – content – based framework for O118 allows in-depth cluster interpretations, particularly for lineages with high-risk combinations of virulence and resistance determinants. Risk assessment can be clearly improved by integrating phylogroup membership with key virulence determinants. Across O118 STEC, we observed phylogroups spanning multiple H-types with diverse Stx profiles and frequent carriage of LEE, highlighting that the O118 serogroup contains multiple lineages with distinct virulence potential (Figure 7, Table S1, Table S3). The consistent association between H-type and *eae-*subtype (e.g. H6/*eae-ι*, H16/*eae-β*, H2/*eae-ε*) provides an additional discriminator that can be used alongside *stx*-subtyping. As reported, a subset of H2-STEC lacked *stx*, consistent with potential secondary ΦStx phage loss [100]. This observation is important for surveillance and laboratory interpretation because *stx*-negative derivatives can complicate routine screening and may be misclassified without phylogenomic context. The described AMR/MDR inventory, including a newly resolved pathogenicity-associated MDR island that harbors loci related to colonization and interbacterial competition, underscores that subsets of O118 STEC infections may exhibit enhanced pathogenic potential and limited therapeutic options.

## Supplementary Material

Table_S4_ST_Achtman_MLST.xlsx

Table_S3_Virulence_and_antimicrobial_resistance_gene_content.xlsx

Figure_S3_Mauve_Identify_Indels_Recom.jpg

O118_Supplemental_Figures_Tables_Legends.docx

Table_S1_Strain_associated_metadata_and_genome_stats.xlsx

Fig_S2_IS_elements.jpg

Fig_S1_ANIclustermap.jpg

Fig_S4_Stx1a_Prophage_Remnants_Mapped.jpg

Table_S2_Prophage_and_mobilome_gene_content.xlsx

## Data Availability

The sequence data sets generated and analyzed in this study have been deposited in the Sequence Read Archive (SRA) and GenBank at NCBI under BioProject PRJNA1103183 (https://www.ncbi.nlm.nih.gov/search/all/?term=PRJNA1103183). Accessions for genomic reads, assembled annotated chromosomes, and plasmids, along with strain-associated metadata, are provided in Table S1. Supplementary tables and figures are available from figshare: (https://doi.org/10.6084/m9.figshare.30194302) [185]. A preprint is available from bioRxiv (https://doi.org/10.1101/2025.04.29.651274) [186].
