# Supplementary figures and images for: Phylogenomic framework and virulence gene boundaries of emerging Shiga toxin-producing Escherichia coli O118 informed by the comprehensive profiling of 359 O118 genomes
Source: Virulence. 2026 May 15;17(1):2672206. doi: 10.1080/21505594.2026.2672206 (PMC13182975; doi:10.1080/21505594.2026.2672206)

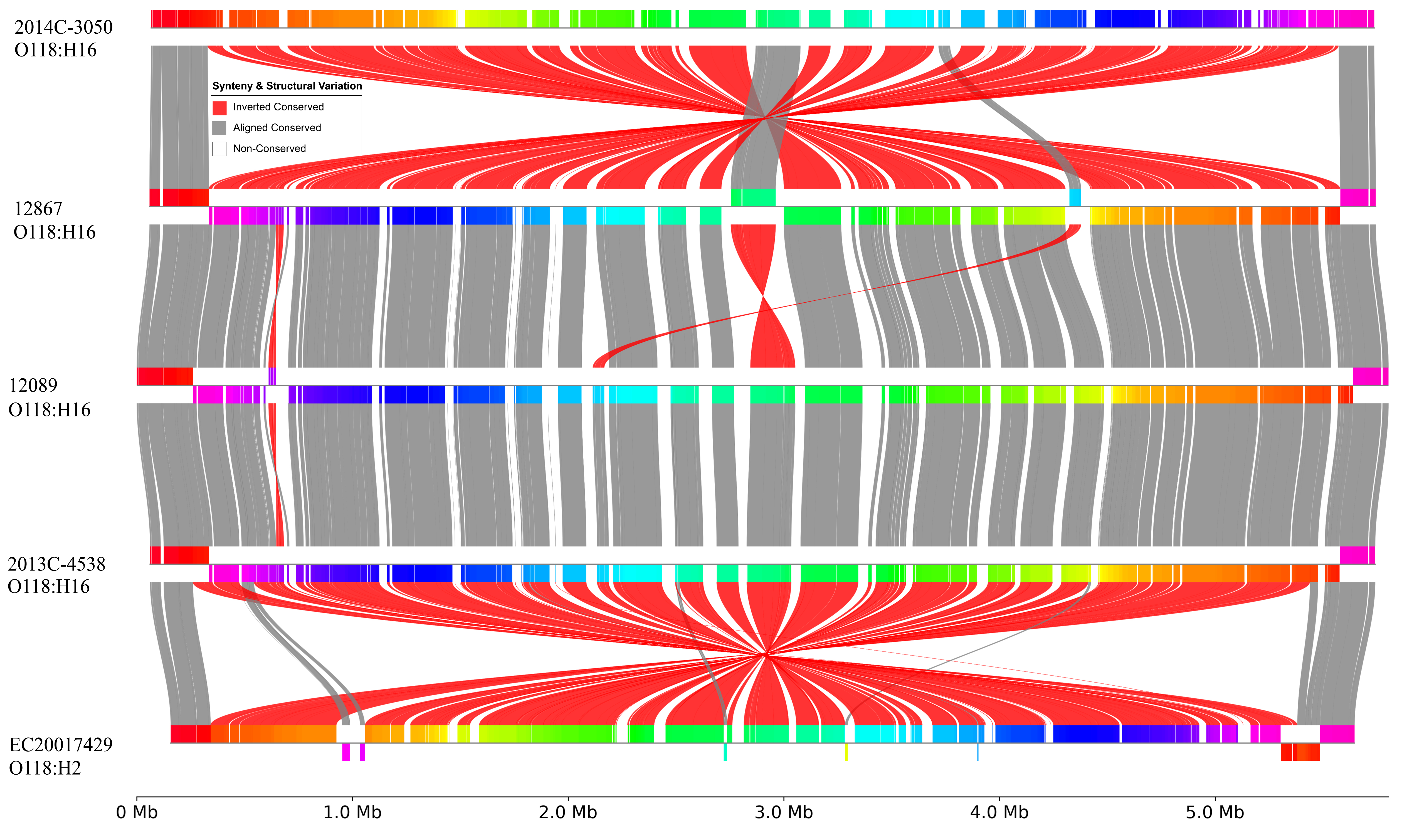

Supplement: Figure_S3_Mauve_Identify_Indels_Recom.jpg [file KVIR_A_2672206_SM2577.jpg]

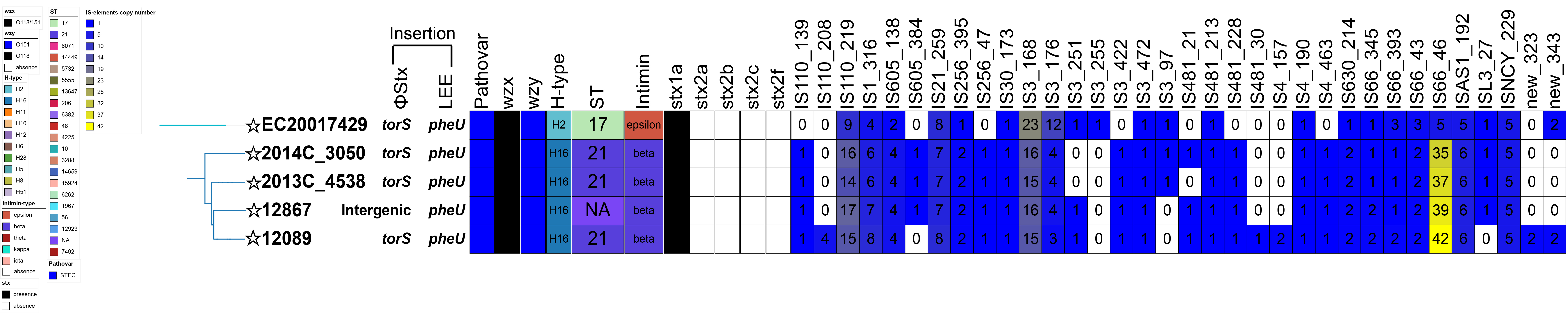

Supplement: Fig_S2_IS_elements.jpg [file KVIR_A_2672206_SM2574.jpg]

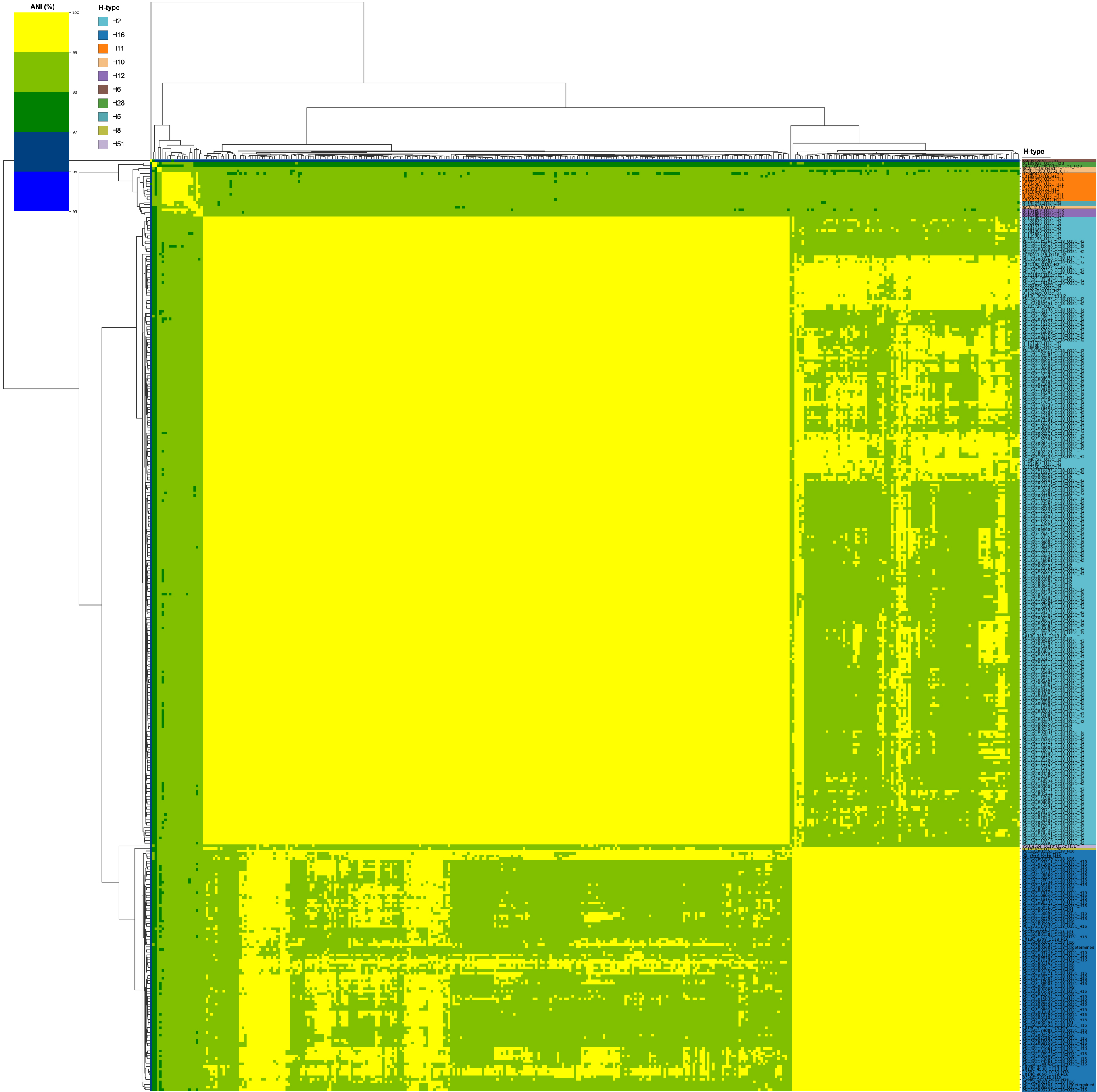

Supplement: Fig_S1_ANIclustermap.jpg [file KVIR_A_2672206_SM2573.jpg]

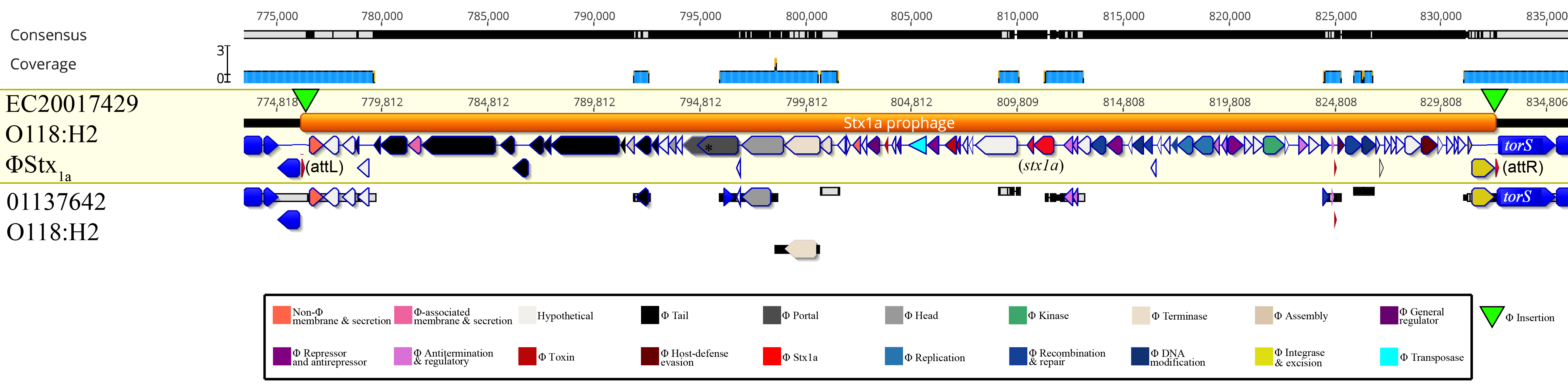

Supplement: Fig_S4_Stx1a_Prophage_Remnants_Mapped.jpg [file KVIR_A_2672206_SM2572.jpg]
