# Supplementary material for: Phylogenomic framework and virulence gene boundaries of emerging Shiga toxin-producing Escherichia coli O118 informed by the comprehensive profiling of 359 O118 genomes
Source: Virulence. 2026 May 15;17(1):2672206. doi: 10.1080/21505594.2026.2672206 (PMC13182975; doi:10.1080/21505594.2026.2672206)
Supplement: O118_Supplemental_Figures_Tables_Legends.docx [file KVIR_A_2672206_SM2576.docx]

**Supplemental Figures**

**FIG S1 Phylogenetic relationship inferred from Average Nucleotide Identities.** UPGMA-clustered distance matrix of ANI values computed by an all-vs-all comparison of the 359 genomes.

**FIG S2 IS elements in the closed STEC O118 chromosomes.** This heatmap shows the prevalence and distribution of the 726 cataloged IS elements closed H2 and H16 O118 STEC, which can be categorized into 16 known families and 40 clusters. As determined by MLST typing, individual strain relationships are also reflected in IS cluster types and copy numbers.

**FIG S3 Comparison of closed O118 genome architectures.** Mauve-comparisons of chromosomes highlighting genomic rearrangements associated with hot spots for recombination.

**FIG S4 Comparison of the ΦStx1a-prophages insertion sites in H2 strains.** BLASTn-based comparison of the ΦStx_1a_ prophage insertion sites in H2 *stx*+/- isolates. The comparisons show that phage remnants were detected in related *stx_1a_* negative phylogroups (H2, *stx-*) with the same phage integrase type at *torS*.

**Supplemental Tables**

**TABLE S1** Strain-associated metadata and genome statistics

**TABLE S2** Predicted prophage and mobilome gene content

**TABLE S3** Predicted virulence and antimicrobial resistance gene content

**TABLE S4** Sequence Types according to the Achtman MLST schema
